# Predictive Value of Neutrophil–Lymphocyte Ratio and Other Inflammation Indices in Febrile Seizures in Children

**DOI:** 10.3390/jcm13175330

**Published:** 2024-09-09

**Authors:** Yakup Söğütlü, Uğur Altaş

**Affiliations:** 1 Ümraniye Training and Research Hospital, Department of Pediatrics, Pediatric Emergency Medicine Clinic, University of Health Sciences, 34764 İstanbul, Türkiye; beyoglu@hotmail.com; 2 Ümraniye Training and Research Hospital, Department of Pediatrics, Pediatric Allergy and Immunology Clinic, University of Health Sciences, 34764 İstanbul, Türkiye

**Keywords:** febrile seizure, inflammation, systemic inflammation response index, systemic immune-inflammation index, pan-immune-inflammation value, neutrophil–lymphocyte–platelet ratio

## Abstract

**Objective:** There is increasing evidence for the effect of inflammation on the etiology of febrile seizure (FS) patients. We aimed to investigate the role of easily accessible inflammatory markers such as the neutrophil–lymphocyte ratio (NLR), systemic immune-inflammation index (SII), systemic inflammation response index (SIRI), neutrophil–lymphocyte–platelet ratio (NLPR), and pan-immune-inflammation value (PIV) in febrile seizure. **Methods:** A total of 300 children, including 100 with febrile convulsions (FS), 100 febrile controls (FCs), and 100 healthy controls (HCs), were included in this retrospective study. The FS group was compared with the FC and HC groups in terms of these inflammatory indexes. **Results:** Between the FS group and the FC group, the neutrophil count was significantly higher in the FS group (*p* = 0.001) and the lymphocyte count was significantly lower (*p* < 0.001). The NLR (*p* < 0.001), SII (*p* < 0.001), SIRI (*p* < 0.001), NLPR (*p* < 0.001), and PIV (*p* < 0.001) were significantly higher in the FS group than in both the FC and healthy control groups. The optimal cut-off values for predicting FS in febrile conditions were 3.59> for NLR, >870.47 for SII, >1.96 for SIRI, 0.96> for NLPR, and >532.75 for PIV. **Conclusions:** The inflammatory indices are inexpensive, easily accessible hematological markers that can contribute to the diagnosis of FS.

## 1. Introduction

Febrile seizures are defined as a type of seizure that occurs in children between 6 and 60 months of age and that is accompanied by a fever of at least 38 °C without central nervous system infection [1]. They are the most common seizures in children younger than 5 years of age. Febrile seizures occur more frequently in males than in females, with a ratio of 1.6:1 [2]. The incidence of febrile seizures in the United States and Western Europe is estimated to be between 2% and 5% [3].

Although numerous studies have investigated FS, the exact pathophysiology remains unclear. Cytokines are among the factors that may play a role in the pathogenesis of FS. Cytokines are mediators of the host’s response to infections and induce fever, leukocytosis, and acute-phase protein synthesis [4].

Most diseases that cause fever do not cause seizures. Although the factors that cause seizures have been investigated, there is no consensus on the pathophysiology. One of the proposed mechanisms in this regard is inflammatory pathway activation and an increase in cytokines. Cytokines are mediators of the host’s response to infections and induce fever, leukocytosis, and acute-phase protein synthesis. Especially in patients with FS, high CSF IL-1β and serum IL-6 levels are observed. However, these cytokines are not routinely used and have high costs. This has paved the way in the search for inexpensive and easily available indices reflecting inflammation, such as the neutrophil–lymphocyte ratio (NLR). Recently, some biomarkers have been used practically in the prediction of certain medical conditions [5]. For example, the NLR was used for predicting the severity of isolated tibial plateau fractures in a study [6]. Another study found comparable results, incorporating the platelet–lymphocyte ratio (PLR) to assess the severity and prognosis of acute spinal cord injuries [7]. Numerous studies in patients with FS have also demonstrated the relationship between FS and NLR. The NLR has also been reported to be an inexpensive diagnostic biomarker for FS, which may also be useful when distinguishing between simple FS and complex FS [8].

The systemic immune-inflammation index (SII), systemic inflammation response index (SIRI), and pan-immune-inflammation value (PIV) are the three new indices for inflammation. These indices are receiving more and more attention every day. The SII is a new index obtained by multiplying the platelet count and the ratio of neutrophils to lymphocytes. Combined ratios, including the systemic immune-inflammation index (SII) and the systemic inflammation response index (SIRI), have demonstrated their utility in predicting outcomes for COVID-19 patients requiring admission to intensive care units [9,10]. In the literature, the SII has been studied in necrotizing enterocolitis [11], myocarditis [12], malignancy [13], fever of unknown cause [14], and neonatal sepsis [15] in pediatric patients.

The SII is calculated by multiplying the SIRI monocyte count by the neutrophil–lymphocyte ratio and reflects increased inflammation and decreased immune response. Although it has been studied mostly in malignancy [16], it is also related to prognosis and clinical outcomes in different areas, such as cardiovascular disease [17], rheumatological disease [18], and acute pancreatitis [19].

The pan-immune-inflammation value has also been studied mostly in malignant patients and is thought to be a prognostic biomarker [20]. It is thought to reflect inflammation and immune status while also being associated with vasculitis [21] and childhood hepatosteatosis [22]. The neutrophil–lymphocyte–platelet ratio (NLPR) is another new inflammatory marker. It has been associated with prognosis in infectious diseases [23,24].

We investigated whether the NLR, SII, SIRI, NLPR, and PIV of febrile children with and without seizures were effective biomarkers in determining the occurrence and types of FS. In addition, we assessed whether they were effective biomarkers for identifying simple and complex FS.

## 2. Materials and Methods

### 2.1. Study Design and Population

For this case–control study, patients between the ages of 0 and 6 years who were admitted to the pediatric emergency department of the University of Health Sciences Ümraniye Training and Research Hospital with febrile convulsions between June 2022 and December 2022 were retrospectively screened from hospital medical records.

**Figure 1:**
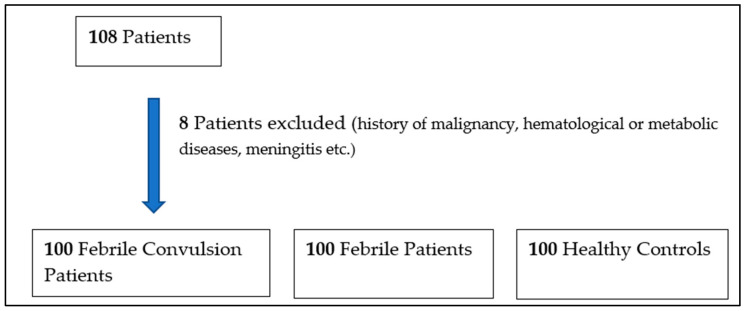
Participants included in the study.

Patients who presented with fever but did not have convulsions, along with healthy children, were also included as healthy control groups. However, children with a previous history of afebrile convulsions; children with structural or developmental central nervous system abnormalities; those with a history of malignancy, hematological or metabolic diseases, or meningitis; those diagnosed with encephalitis, biphasic encephalopathy, acute encephalopathy with biphasic seizures and late reduced diffusion (AESD), or febrile convulsive encephalopathy; and those with steroid and antiepileptic use were excluded from the study. According to these criteria, 8 patients were excluded from the study and 100 febrile convulsion patients were included. A further 100 febrile patients and 100 healthy controls were included in the study by scanning the records in terms of age and sex (Figure 1).

Ethics committee approval was obtained by the Istanbul Health Sciences University Ümraniye Training and Research Hospital Clinical Research and Ethics Committee (Date: 23 February 2023, Number: B.10.1.THK.4.34.H.GP.0.01/49).

### 2.2. Measures

Age and gender percentages were evaluated between the groups. The etiology of fever in patients who experienced it was obtained from the medical records. Additionally, the number of convulsions each patient experienced and the type of convulsions (either simple or complex) were determined. Electroencephalography (EEG) and magnetic resonance imaging (MRI) results of patients with complex seizures were recorded. In the study, the term “febrile seizures” was used in accordance with the international terminology guidelines provided by the International League Against Epilepsy (ILAE) [25]. Febrile seizures are defined as seizures occurring in the context of fever without an underlying central nervous system infection or other specific cause. We distinguished between simple febrile seizures and complex febrile seizures. Simple febrile seizures are typically brief and occur once within a 24-h period. However, complex febrile seizures have prolonged duration (more than 15 min) or are characterized by multiple seizures occurring within 24 h, featuring focal neurological signs.

Hemoglobin, white blood cell (WBC) count, neutrophil, lymphocyte, monocyte, platelet count, mean platelet volume (MPV), mean corpuscular volume (MCV), red blood cell distribution width (RDW), plateletcrit (PCT), and C-reactive protein (CRP) values were recorded during the first admission to the emergency room before treatment.

In the laboratory, Mindray BC-6200 as a hematology autoanalyzer (High-tech IndustrialPark, Nanshan, Shenzhen 518057, P. R. China) was used. The neutrophil–lymphocyte ratio was obtained by dividing the absolute number of neutrophils by the absolute number of lymphocytes. The systemic immune-inflammation index was defined as neutrophil/lymphocyte ratio × platelet. Systemic inflammatory response index was defined as SIRI = (neutrophil count × monocyte count)/lymphocyte count. The neutrophil lymphocyte platelet ratio was calculated as (NLPR) = (Neutrophilx100)/(lymphocyte × platelet). Pan-immune-inflammation value (neutrophil x platelet x monocyte)/lymphocyte was also calculated.

### 2.3. Statistical Analysis

The normality of the distribution of continuous variables was examined using visual (histograms and probability plots) and analytical methods (Kolmogorov–Smirnov/Shapiro–Wilk tests). Continuous variables were presented as mean ± standard deviation; continuous variables without normal distribution were presented as median (minimum-maximum). Categorical data were presented as numbers (n) and percentages (%). Chi-square test was used for comparison of categorical variables. For comparisons of continuous variables, the Mann–Whitney U test was used for non-normally distributed data in binary groups. T test was performed for continuous variables that fit the normal distribution.

Receiver Operating Characteristic (ROC) curve analysis was utilized to calculate the optimal cut-off values, sensitivity, and specificity of NLR, SII, SIRI, PIV, and NLPR. The optimum cutting value was determined by the Youden index. A *p* value below 0.05 was considered as the level of statistical significance. SPSS 22 (IBM Corp, SPSS, Armonk NY, USA) program was used for data recording and statistical analysis.

## 3. Results

This study included 300 children, comprising 100 febrile convulsions, 100 febrile children, and 100 healthy control groups. The mean age of the FS group was 27.9 ± 8.5 months, the febrile control group was 26.5 ± 10 months, and the control group was 29.9 ± 9.5 months. There was no significant difference between the groups in terms of age (*p* > 0.05). There was no significant difference between the groups in terms of sex in the FS and FC groups (*p* > 0.05). The most common etiology of fever in both the FS group and the febrile control group was upper respiratory tract infection (Table 1).

It was the first seizure for 74% of the cases, the second for 15%, the third for 9%, the fourth for 1%, and only 1% had a fifth seizure. Moreover, 90% were simple seizures and 10% were complex seizures. The mean age of patients with simple seizures was 28 ± 12.2 months and the mean age of patients with complex seizures was 26.9 ± 15.6 months; therefore, the mean age was similar (*p* = 0.512). The EEG and MRI results of all patients with complex seizures were normal. The mean time from seizure to sample collection was 40 ± 22 min. Median WBC values were 13.1 in the FS group and 13.5 in the febrile control group. While there was no significant difference between these two groups in terms of WBC (*p* = 0.722), there was a difference in leukocyte subgroups. In FS, neutrophil counts were significantly higher (*p* = 0.001) than febrile control and lymphocyte counts were significantly lower (*p* < 0.001). Median neutrophil values were 8.8 and 7.1 in FS and febrile control, respectively, while median lymphocyte values were 2.5 and 4.2, respectively.

Median monocyte values were 1.02 in FS and 1.0 in febrile controls, respectively. There was no significant difference between the groups (*p* = 0.708). Platelet values were significantly lower in FS than in the febrile control group (*p* = 0.032) (Table 1).

In terms of hematologic and inflammatory parameters, WBC, neutrophil, monocyte, NLR, SII, SIRI, PIV, and NLPR values were significantly higher when the FS group was compared with the healthy control group, while lymphocyte, hemoglobin, and platelet values were significantly lower. When FS was compared with the febrile control group in terms of hematologic and inflammatory parameters, NLR, SII, SIRI, PIV, and NLPR values were found to be significantly higher in the FS group (Table 1).

When the inflammatory parameters of those with simple and complex seizures were compared, no significant difference was observed. The median values of NLR were 3.8 (0.5–21.3) in patients with simple seizures and 2.8 (1.4–11) in patients with complex seizures (*p* = 0.749). SII median values were 1048 (114–4880) in patients with simple seizures and 772 (288–3021) in patients with complex seizures (*p* = 0.420). SIRI median values were 3.2 (0.4–21.6) in patients with simple seizures and 3.3 (0.9–21.6) in patients with complex seizures (*p* = 0.839). The median PIV values were 970 (104–7866) in patients with simple seizures and 785 (172–5921) in patients with complex seizures (*p* = 0.940). When the inflammatory parameters of patients with recurrent seizures were compared with those with first-time seizures, there was no significant difference in terms of WBC, neutrophil, lymphocyte, monocyte, NLR, SII, SIRI, PIV, and NLPR (*p* > 0.05 for each).

**Figure 2:**
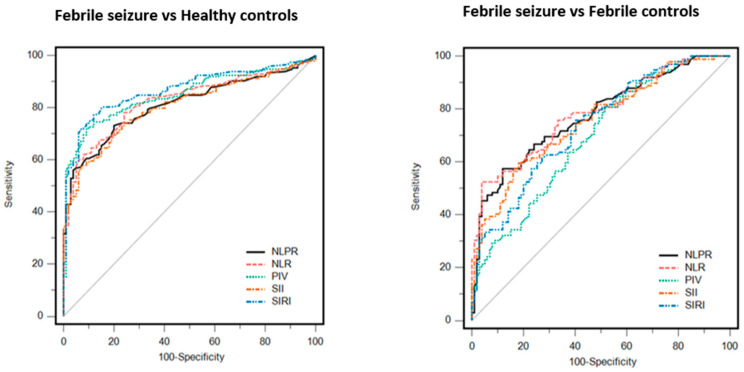
Receiver operating curve results of inflammatory indices for the distinction between FS and FC and for the distinction between FS and HC. NLR: neutrophil/lymphocyte ratio. SIRI: systemic inflammation response index. SII: systemic immune-inflammation index. PIV: pan-immune-inflammation value. NLPR: neutrophil/lymphocyte/platelet ratio.

We determined the ROC curve of inflammatory markers to predict seizure in febrile patients (Table 2, Figure 2). The area below the ROC curve in predicting severe febrile seizure was 0.774 (*p* < 0.001) for Area Under Curve (AUC) NLPR, 0.782 (*p* < 0.001) for NLR, 0.755 (*p* < 0.001) for SII index, 0.737 (*p* < 0.001) for SIRI, and 0.692 (*p* < 0.001) for PIV. NLR had the highest AUC and the highest specificity. The optimal cut-off, sensitivity, and specificity values of all inflammatory markers for the diagnosis of FS are shown in Table 2. In addition, the ROC curve of inflammatory markers for the separation of FS and HC is shown in Table 2 and Figure 2.

## 4. Discussion

The main finding of this study is that there is a relationship between febrile seizure and the inflammatory markers of NLR, SII, SIRI, NLPR, and PIV. The first publication revealing the relationship between FS and CBC parameters in children was reported in 2014. This study demonstrates that NLR and red blood cell distribution width (RDW) can be simple and effective determinants in differentiating FS types [26]. Later studies have suggested that high NLR levels are associated with FS [27,28,29]. Hosseini and his colleagues recently published a meta-analysis examining the relationship between febrile seizure and NLR. This meta-analysis included 17 studies including 1919 children with FS and 1079 febrile controls. As a result, it was reported that both the NLR levels of children with FS were higher compared to febrile controls, and the NLR levels of the simple FS group were significantly higher than those of complex FS patients [8]. In contrast, some studies have not found NLR, PLT, and monocyte values to be useful in differentiating between simple febrile seizure (SFS) and complex febrile seizure (CFS) [30]. Although Pooja et al. found the NLR rate to be high in CFS in their study in which they included 100 FS and 100 febrile control patients, the difference between it and simple seizures was not significant. The NLR rate of children with FS was found to be significantly higher than the control group [31].

In the 10 studies included in the meta-analysis of Hosseini et al., febrile patients were included as a control group. In different studies, the mean NLR level in the FS group ranged between 1.84 and 4.73, while the mean NLR level in febrile control patients ranged between 0.91 and 3.72 [8]. In our study, the median value of NLR was found to be 3.6 in the FS group, 1.5 in the FC group, and 0.8 in the healthy control group. NLR values were found to be significantly higher in the FS group than in both the FC group and healthy controls. However, there was no significant difference between simple and complex seizures in terms of NLR, SII, SIRI, PIV, and NLPR. Similarly, there was no difference between the first seizure and recurrent seizures in terms of the same inflammatory indices.

The NLR is an index that reflects inflammation. In the development of epilepsy, there is increasing emphasis on the role of innate and adaptive immune responses through mediators such as cytotoxic T cells, complement activation, and reactive microglia [32]. The neutrophil increase seen in patients with FS is thought to be due to the effect of elevated adrenaline and cortisol levels resulting from both an increase in cytokines and heightened sympathetic activity [29]. It has been suggested that muscle activity during convulsions also contributes to an increase in neutrophil counts [33].

The SII value evaluated in this study was obtained by NLR and platelet multiplication. To the best of our knowledge, there is no study evaluating SII in FS patients, but there is a study evaluating platelet levels with FS. In a study by Tang et al., which investigated the relationship between febrile seizures and platelet levels, the platelet levels of FS patients were found to be significantly lower than those in both the febrile control and healthy control groups. In addition, this study found platelet counts to be significantly lower in complex seizures than in simple seizures. Platelet counts were lower in patients with FS recurrence than in those without relapse [32]. It has been suggested that low platelet levels in these patients are associated with the activation and aggregation of platelets by inflammatory mediators that occur during infection. In line with this, our study has also illustrated the PLT levels to be lower in the FS group than in the controls. The elevation of SII in the FS group appears to be due to the increase in NLR levels.

When we evaluate SIRI index in our study, we demonstrated that it was higher in FS patients than in FC patients. SIRI is a parameter obtained by multiplying the monocyte count with the NLR. In our study, we found no significant difference in monocyte values between FS and FC patients. We interpreted a significant increase in SIRI values in FS patients caused by the contribution of NLR elevation.

To the best of our knowledge, the relationship with NLPR in FS patients has not been investigated. In our study, NLPR values were found to be significantly higher in the FS group than in both the FC group and healthy controls. Since platelet value is the denominator in the calculation of this parameter as opposed to the SII index, low platelets in FS patients seem to contribute to the increase in NLPR levels. Although this suggests that the effect of NLPR may be stronger than NLR, the AUC value was found to be higher in NLR in ROC analysis.

PIV, another inflammatory index we investigated in our study, is obtained by dividing the multiplication of neutrophils, monocytes, and platelets by the number of lymphocytes. The fact that the number of monocytes was not different between FC and FS patients and that the platelet count was lower in FS patients than in FC patients seems to have left the PIV index behind the NLR index. Although PIV values are significantly higher in FS patients than in FC and HC, the contribution of NLR to this elevation seems to be high. In the ROC analysis, the PIV value had the lowest AUC value in differentiating FS patients from FC patients.

### Limitations and Strengths

This study is the first to investigate the relationship between FS and inflammatory indices such as SII, SIRI, NLPR, and PIV. Although we found that these indices were not useful in differentiating between seizure types, our research contributes novel insights into the potential role of systemic inflammation in febrile seizures. This is the strength of this study. In addition, the study’s focus on easily accessible and cost-effective biomarkers holds practical value for clinical settings.

In addition to the strengths, there are also some limitations to consider. The study’s single-center design may limit the generalizability of the findings, and its retrospective nature prevented us from evaluating changes in proinflammatory cytokines, which could have provided further understanding of the pathophysiology. Additionally, we only assessed inflammatory parameters at the time of emergency department admission, without accounting for potential fluctuations over time. While it is well known that the neutrophil-to-lymphocyte ratio varies with age during childhood, particularly in children under one year of age, we did not perform an age-stratified analysis in our study. This situation may be considered as another limitation of the study, as it may have a potential impact on the results.

## 5. Conclusions

In conclusion, NLR, SII, SIRI, NLPR, and PIV inflammatory indices are inexpensive, easily accessible, and effective indices in the development of febrile seizures. However, these inflammatory indices were not found to be useful in differentiating the types of FS. While our study provides valuable insights, it underscores the need for further research. Large multicenter studies are required to clarify the complex relationship between inflammation and FS and to explore potential therapeutic targets that could improve patient outcomes. Additionally, prospective studies that monitor inflammatory markers over time may provide a more dynamic understanding of their role in the pathophysiology of FS.

## Figures and Tables

**Table 1:** Comparison of demographics, laboratory characteristics, and inflammatory indices of the groups included in the study.

	Febrile Seizure (FS) Group	Fever Control (FC) Group	Healthy Control (HC)	*p* Value	
				FS Group vs. FC Group	FS Group vs. HC Group
Age (months), mean ± SD	27.9 ± 8.5	26.5 ± 10	29.9 ± 9.5	0.440	0.373
Gender (male, %)	65	62	50	0.657	0.022
Fever etiology, n (%)				0.052	
Upper rsp.tract inf.	89	75			
Gastroenteritis	5	5			
Pneumonia	4	17			
Urinary tract inf.	1	2			
Otitis	1	1			
WBC, ×10^9^/L, median (min–max)	13.1 (3.6–35.5)	13.5 (2.3–27.5)	8.7 (5.5–10.5)	0.722	<0.001
Neutrophil, ×10^9^/L, median (min–max)	8.8 (1.7–26.5)	7.1 (0.6–17.8)	3.5 (1–7.8)	0.001	<0.001
Lymphocyte, ×10^9^/L, median (min–max)	2.5 (0.4–16.6)	4.2 (0.7–17.3)	4.3 (1.8–8.8)	<0.001	<0.001
CRP (mg/L), median (min-max)	10.7 (0.2–104)	19.3 (0.9–280)		<0.001	
Monocytes, ×10^9^/L, median (min–max)	1.02 (0.4–2.5)	1 (0.2–2.7)	0.58 (0.2–2)	0.708	<0.001
Hemoglobin (g/dL), median (min-max)	11.2 (6.6–13.2)	11.2 (7.4–15.5)	11.8 (9.8–14.7)	0.309	<0.001
MCV (fL), mean ± SD	76.2 ± 6.8	76.3 ± 6.4	77.9 ± 5	0.939	0.043
RDW (fL), median (min–max)	14 (11.7–18.5)	14.3 (12.9–21.7)	13.7 (12.1–20.2)	0.199	0.132
Platelet, ×10^9^/L, median (min–max)	284 (137–560)	321 (57–966)	336 (141–509)	0.032	0.001
MPV (fL), median (min–max)	8 (6.7–11.8)	8.2 (6.6–12.6)	8.4 (6.7–10.7)	0.058	0.008
PCT (%), median (min-max)	0.24 (0.1–0.5)	0.26 (0.07–0.8)	0.3 (0.13–0.61)	0.007	<0.001
NLR, median (min–max)	3.6 (0.5–21.3)	1.5 (0.1–5.8)	0.8 (0.1–2.9)	<0.001	<0.001
SII median (min–max)	1003.5 (114–4880)	482.2 (17.3–1870)	272.1 (54.6–943.2)	<0.001	<0.001
SIRI median (min–max)	3.24 (0.4–21.6)	1.6 (0.03–13.1)	0.5 (0.06–4.7)	<0.001	<0.001
PIV median (min–max)	910.3 (103.8–7866.4)	565.1 (4.1–2431.5)	151.0 (20.9–1778.5)	<0.001	<0.001
NLPR median (min–max)	1.1 (0.1–9.4)	0.4 (0.03–4.7)	0.2 (0.03–1)	<0.001	<0.001

NLR: neutrophil/lymphocyte ratio. SIRI: systemic inflammation response index. SII: systemic immune-inflammation index. PIV: pan-immune-inflammation value. NLPR: neutrophil/lymphocyte/platelet ratio. CRP: C-reactive protein. RDW: red blood cell distribution width. MCV: mean corpuscular volume. MPV: mean platelet volume. PCT: plateletcrit.

**Table 2:** Receiver operating curve analysis results of inflammatory indices for the distinction between FS and FC and for the distinction between FS and HC.

	ROC Curve Analysis			Statistical Diagnostic Measures			
	AUC (95% CI)	*p*-Value	Optimal Cut-Off	Sensitivity	Specificity	PPV	NPV
FS vs. HC							
NLPR	0.811 (0.762–0.854)	<0.001	>0.38	73.23 (66.5–79.3)	79.80 (70.5–87.2)	87.9 (82.9–91.5)	59.8 (53.7–65.7)
NLR	0.825 (0.777–0.867)	<0.001	>1.14	78.28 (71.9–83.8)	75.76 (66.1–83.8)	86.6 (81.9–90.2)	63.6 (56.7–69.9)
SII	0.804 (0.755–0.848)	<0.001	>451.64	69.70 (62.8–76.0)	80.81 (71.7–88)	87.9 (82.8–91.7)	57.1 (51.4–62.7)
SIRI	0.868 (0.824–0.905)	<0.001	>1.02	77.27 (70.8–82.9)	87.88 (79.8–93.6)	92.7 (88.2–95.6)	65.9 (59.7–71.6)
PIV	0.850 (0.804–0.888)	<0.001	>396.21	72.22 (65.4–78.3)	90.91 (83.4–95.8)	94.1 (89.4–96.8)	62.1 (56.4–67.4)
FS vs. FC							
NLPR	0.774 (0.709–0.830)	<0.001	>0.96	57.58 (47.2–67.5)	87.88 (79.8–93.6)	82.6 (73.1–89.2)	67.4 (61.9–72.5)
NLR	0.782 (0.718–0.838)	<0.001	>3.59	52.53 (42.2–62.7)	95.96 (90–98.9)	92.9 (83–97.2)	66.9 (62.1–71.4)
SII	0.755 (0.689–0.813)	<0.001	>870.47	59.60 (49.3–69.3)	81.82 (72.8–88.9)	76.6 (67.7–83.7)	66.9 (61–72.4)
SIRI	0.737 (0.670–0.797)	<0.001	>1.96	75.76 (66.1–83.8)	59.60 (49.3–69.3)	65.2 (59–70.9)	71.1 (62.6–78.3)
PIV	0.692 (0.623–0.756)	<0.001	>532.75	80.81 (71.7–88)	48.48 (38.3–58.7)	61.1 (55.9–66)	71.6 (61.6–79.9)

NLR: neutrophil/lymphocyte ratio. SIRI: systemic inflammation response index. SII: systemic immune-inflammation index. PIV: pan-immune-inflammation value. NLPR: neutrophil/lymphocyte/platelet ratio.

## Data Availability

The original contributions presented in this study are included in the article; further inquiries can be directed to the corresponding author.
